# A Diagnostic Challenge: Periorbital or Orbital Cellulitis?

**DOI:** 10.7759/cureus.48439

**Published:** 2023-11-07

**Authors:** Sarayu Vanga, Anjali R Daniel, Murdoc B Gould, Sidhartha R Ramlatchan, Latha Ganti

**Affiliations:** 1 Biomedical Sciences, University of Central Florida, Orlando, USA; 2 Biology, Emory University, Atlanta, USA; 3 Chemistry, Rollins College, Orlando, USA; 4 Chemistry, Drexel University, Philadelphia, USA; 5 Medical Sciences, The Warren Alpert Medical School of Brown University, Providence, USA; 6 Emergency Medicine & Neurology, University of Central Florida College of Medicine, Orlando, USA

**Keywords:** eye discharge, post septal cellulitis, preseptal cellulitis, orbital cellulitis, periorbital cellulitis

## Abstract

The authors present the case of a 39-year-old male who presented to the hospital with worsening eye pain, swelling, and blurred vision of the left eye. His symptoms grew worse despite initial over-the-counter medication and a trip to the urgent care center. A physical exam was concerning for a possible orbital cellulitis given the appearance of the eye and the amount of discomfort, as well as their immunocompromised status, necessitating imaging and workup to confirm the diagnosis. The patient was ultimately diagnosed with periorbital cellulitis and bacterial conjunctivitis, and he received intravenous antibiotics for treatment. This case underscores the importance of a comprehensive diagnostic approach to managing ocular infections.

## Introduction

Periorbital cellulitis, also referred to as pre-septal cellulitis, is an infection around the eyelid affecting the surrounding skin and tissue in the location anterior to the orbital septum [1, 2]. It is often caused by trauma or sinusitis and rarely leads to severe complications [3]. A similar condition, orbital cellulitis, also known as post-septal cellulitis, presents quite similarly to preorbital cellulitis, which occurs posterior to the orbital septum but is more concerning as it can lead to sight-threatening complications [4,5]. The infection can penetrate the intraconal structures, leading to vascular complications [3]. Signs of orbital cellulitis may include visual loss, ophthalmoplegia, pain in eye movement, and proptosis [3, 5, 6]. By contrast, those diagnosed with periorbital cellulitis often present characteristics of unilateral swelling of the eyelid, superficial pain, erythema, and edema [5]. Common causes of this condition include superficial scratches (trauma) or insect bites around the eye, which eventually lead to infection. It is slightly more prevalent in men and in the spring, likely related to activities that lead to eye trauma [6]. Patients may experience normal to slightly impaired vision and feelings of discomfort when blinking. The common bacteria seen in periorbital cellulitis are *Streptococcus pneumonia*, *Streptococcus anginosus*, and occasionally other streptococci and anaerobes [7]. Antibiotics remain the mainstay for ocular bacterial infections.

## Case presentation

A 39-year-old Caucasian male presented with a history of eye pain and swelling for two weeks prior to his ED visit. He reported left eye pain, redness, and swelling. He initially used over-the-counter eye drops, which provided temporary relief, but he began having irritation in the eye again three days prior. He denied any eye trauma, insect bites, or food getting stuck in the eye that may have triggered the irritation. He went to an urgent care center two days prior and was prescribed antibiotic eye drops, which he did not obtain. His medical history was significant for mood disorder and human immunodeficiency virus (HIV), for which he was taking Descovy® (emtricitabine and tenofovir alafenamide). He was a daily smoker and had no medication allergies.

**Figure 1:**
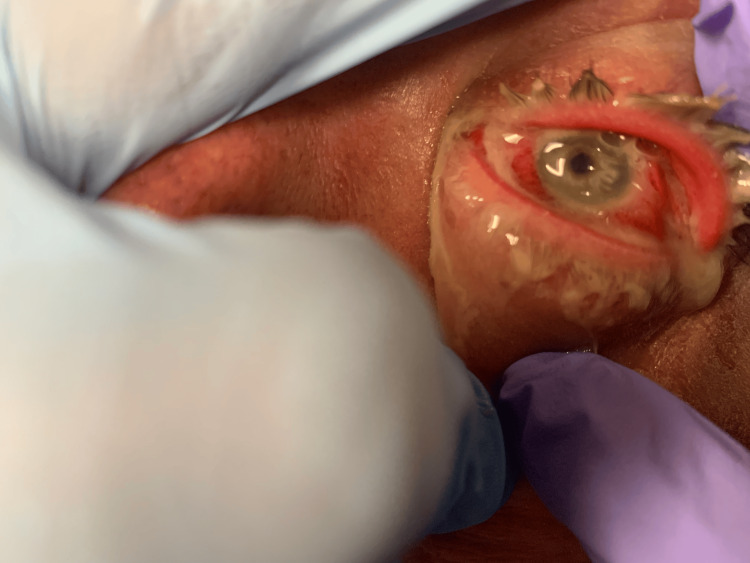
A clinical photograph of the patient's eye

The patient had normal vital signs: a temperature of 98.4°F, a pulse rate of 76 beats per minute, a respiratory rate of 15-18 breaths per minute, and a blood pressure of 133/82 mmHg. He had a normal oxygen saturation of 95% in room air. A physical exam demonstrated 20/20 visual acuity in the right eye but only 20/200 on the left. Left eye examination revealed a matted shunt, photophobia upon assisted opening, and a yellowish eye discharge (Figure 1).

The pupillary examination did not reveal a relative afferent pupillary defect. Extraocular movements in the left eye caused the patient discomfort. Lateral gaze on the left was limited, likely secondary to swelling. A slit-lamp exam revealed a hazy cornea, chemosis, subconjunctival hemorrhages, and an abundance of mucoid discharge. Due to the haziness of the cornea, the funduscopic exam was unable to be completed. Intraocular pressure in the right eye was 20 cmH_2_O (normal) and 25 cmH_2_O on the left (mildly elevated). His neck was supple, without meningismus. The remainder of the physical exam was unremarkable. The laboratory analysis was unremarkable, including a normal white blood cell count. Differential diagnoses included dacryoadenitis, idiopathic orbital inflammation, autoimmune disease, and orbital mass. The patient was admitted to the hospitalist service for broad-spectrum intravenous antibiotics and imaging of the orbits, as it was not clear from the clinical presentation whether this was periorbital or orbital cellulitis.

**Figure 2:**
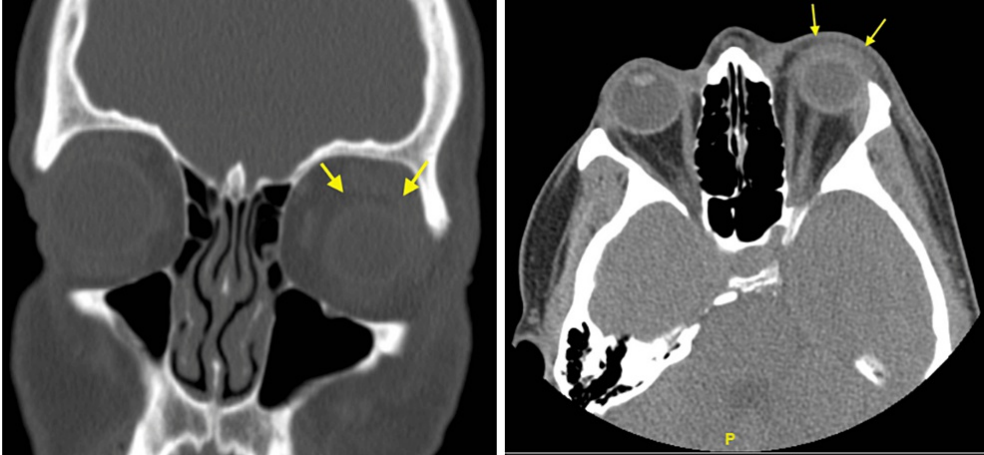
Coronal and axial CT views of the orbits; the arrows denote periorbital edema and soft tissue swelling.

An axial CT scan of the orbits was performed without intravenous contrast. Images of the coronal and sagittal reconstruction were obtained, and a radiation dose optimization technique was utilized for this scan. The CT scan demonstrated left periorbital swelling, an intact globe, and no proptosis. The extraocular muscles and optic nerve sheath complexes were unremarkable. The orbital fat was preserved, and there was no mass. There was left periorbital soft tissue swelling with an enhancement consistent with periorbital cellulitis. The examination showed no evidence of orbital cellulitis, abscess, or mass (Figure 2).

**Figure 3:**
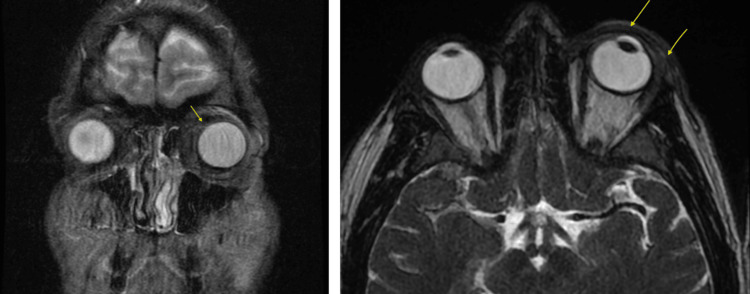
Coronal and axial MRI views of the orbits; the arrows denote periorbital edema and soft tissue swelling.

Based on the CT result, the patient was diagnosed with periorbital cellulitis and bacterial conjunctivitis due to no evidence of orbital cellulitis. He was given intravenous vancomycin and ceftriaxone. He was continued on Descovy® for HIV prevention. An MRI scan performed three days later confirmed left periorbital soft tissue swelling with enhancement consistent with pre-septal cellulitis (Figure 3).

The patient was discharged and advised to follow up with the ophthalmology department in three days. He was also advised to follow up with the infectious disease department in one to two weeks to evaluate for further need for antibiotics. At the two-week primary care follow-up, the patient’s symptoms had resolved.

## Discussion

The treatment of periorbital cellulitis can vary based on each case and the severity of the condition. It is very important to distinguish periorbital from orbital cellulitis. The septum is the separation between the superficial and deeper structures of the eye; they're also known as pre-septal and post-septal cellulitis, respectively. Pre-septal (periorbital) cellulitis typically does not present with eye pain or vision loss, in contrast to orbital cellulitis.

In this case, our patients had several signs of orbital cellulitis, which was confusing. He also had a history of HIV, which put him at higher risk for infection. For this reason, he was managed conservatively, and dedicated orbital imaging was performed. The physician should consider the medical history and medications that the patient is currently taking before prescribing an antibiotic for periorbital cellulitis [3, 8]. The general recommendation for treatment is clindamycin or trimethoprim-sulfamethoxazole with amoxicillin-clavulanic acid due to its efficiency in treatment and methicillin-resistant *Staphylococcus aureus* (MRSA) control purposes [1]. An antibiotic with beta-lactam is recommended if the patient is not immunized with *Haemophilus influenzae* [1, 8]. If an abscess is present, it should be incised and drained. [3].

## Conclusions

Pre-septal (periorbital) and post-septal (orbital) cellulitis are two distinct entities that can be differentiated by their anatomical location relative to the orbital septum, clinical presentation, and potential for serious complications. Post-septal cellulitis typically requires intravenous antibiotics and sometimes surgical intervention, whereas pre-septal cellulitis can often be treated with oral antibiotics on an outpatient basis. Sometimes, as in the case of our patient presented here, symptoms can overlap, which can confound the diagnosis. Imaging studies such as CT or MRI are crucial in differentiating between the two in this case.
